# Potential of Using Shear Wave Elastography in the Clinical Evaluation and Monitoring of Changes in Masseter Muscle Stiffness

**DOI:** 10.1155/2020/4184268

**Published:** 2020-11-12

**Authors:** Cyprian Olchowy, Mieszko Więckiewicz, Luca Maria Sconfienza, Mateusz Łasecki, Piotr Seweryn, Joanna Smardz, Sylwia Hnitecka, Marzena Dominiak, Anna Olchowy

**Affiliations:** ^1^Department of Oral Surgery, Wroclaw Medical University, Wroclaw, Poland; ^2^Department of Experimental Dentistry, Wroclaw Medical University, Wroclaw, Poland; ^3^IRCCS Istituto Ortopedico Galeazzi, Milano, Italy; ^4^Dipartimento di Scienze Biomediche per la Salute, Università degli Studi di Milano, Milano, Italy; ^5^Department of Radiology, Wroclaw Medical University, Wroclaw, Poland

## Abstract

The study aimed to evaluate masseter muscle stiffness in adult healthy volunteers referred to a massage treatment and also to investigate whether shear-wave elastography can be used to monitor the effect of massage on the masseter muscle. The study included 21 healthy volunteers, who were subjected to a 30-minute massage of the masseter muscle. Muscle stiffness was measured by shear-wave elastography before and directly after the massage. Pain during the massage was assessed using the visual analogue scale (VAS). The data of 20 patients (one excluded due to severe pain) with a median age of 34.5 years were analysed. The stiffness values were 11.46 ± 1.55 kPa before and 8.97 ± 0.96 kPa after the massage (*p* < 0.0001). The mean drop was 2.49 ± 1.09 kPa. The greatest decrease was observed in people with higher elasticity values before the massage (*r* = 0.79; *p* < 0.0001). The median intensity of pain was 7.2 (range: 6–9.5). We concluded that shear-wave elastography is a sensitive tool to monitor changes in the stiffness of the masseter muscle.

## 1. Introduction

The masseter muscle constitutes a part of the stomatognathic system. Inappropriate functioning and stiffness of the masseter muscle may lead to many pathologies, including myalgia, myofascial pain, and disordered function [1]. Furthermore, it can also be an aesthetic defect associated with a change of the contours of the face [2]. Increased stiffness of the masseter muscle is observed in many pathologies. Patients with temporomandibular disorders (TMD) often complain about pain in the masseter muscle, as well as its hypertrophy, which clinically manifests with increased stiffness and tension [3, 4]. In patients with fibromyalgia and also those with myofascial pain syndrome, specific biochemical pathways may be responsible for the increased stiffness and tenderness of affected muscles [5]. Moreover, exposure to emotional stress contributes to increased muscle tension [6, 7]. Clinicians highlight the role of addressing increased stiffness and tonus of the masseter muscle as part of the conservative treatment of TMD [6].

Several methods of assessing muscle stiffness have been developed. However, none of them have been introduced into routine clinical practice. Myotonometry has been found to be reliable for measuring muscle stiffness in both larger muscles and smaller muscles, but it is not common for the assessment of dysarthria and TMD [8]. Electromyographic evaluation was also reported to be useful in monitoring the treatment of TMD [9]. Moreover, a portable muscle hardness meter has been used in clinical research [10], and shear-wave elastography can be seen to be a very promising method. Elastography has been validated in studies using phantoms of known hardness and has also been compared to other methods [11, 12]. Results of studies on other organs show that agreement between radiologists is statistically significant [13]. However, despite promising results obtained in studies on healthy volunteers [14, 15], this method still needs to be tested on patients with increased muscle tonus (e.g. suffering from TMD) and those undergoing interventions aimed at reducing muscle stiffness [16].

The aim of this study was to evaluate masseter muscle stiffness in adult healthy volunteers subjected to massage treatment and also to investigate whether shear-wave elastography can be used to monitor the effect of massage on the masseter muscle.

## 2. Materials and Methods

The study included 21 adult healthy volunteers. All of the participants underwent a 30-minute massage session applied to both masseters. Patients who had any neuromuscular disorders, malignancy, local and/or general contraindications for massage, symptoms of TMD, were pregnant, had reported a history of TMD or neuromuscular disorders, or were on muscle relaxants and/or other drugs affecting the muscles (and/or pain killers) were excluded from the study. Generally healthy, adult patients who gave informed written consent were included in the study. The study was approved by the Bioethical Committee at the Wroclaw Medical University (KB, 592/2018).

During the therapy of deep muscle tissue, massage using the following techniques was performed: transverse friction movements of muscle fibers and release techniques of trigger points. The muscle trigger points therapy was conducted by exerting increasing pressure using the tip of the index finger and the thumb until relaxation of the muscle or a significant reduction in pain was perceived by the patient. Next, the pressure was increased to the next pain threshold. This was repeated until the patient perceived no pain. The transverse friction of muscle fibers was conducted for about 2 minutes or until the pain subsided. Similar massage techniques were reported in the literature [17, 18]. The massage was conducted by a physiotherapist with a 5-year experience in physiotherapy of the masticatory muscles.

Masseter muscle stiffness was measured with shear-wave elastography before and immediately after the massage using the Aixplorer Ultimate device (SuperSonic Imagine, Aix-en-Provence, France) with a high-frequency linear probe SL 18–5 (5–18 MHz). On each examination, elastic Young's modulus was recorded, named in this study as an elasticity value and expressed in kPa [16, 19–21]. The patients were examined in a supine, relaxed, and comfortable position. They were advised to relax, not to bite down, and refrain from swallowing during the examination. A small amount of ultrasound gel was used to eliminate the air between the patient's skin and the probe. Scanning was carried out without compressing the examined tissues. The ultrasound probe was placed parallel to the long-axis of the masseter muscle in the middle of the muscle belly where the volume of the fibers is the highest. The authors used a Region of Interest (ROI) of 4 mm diameter for all measurements on all the patients. Three measurements were taken from each muscle, and means of those measurements were analysed. Elastography examinations were performed by a radiologist with seven years of experience. Additionally, all the participants were asked to grade their perceived intensity of pain during the massage and also their perceived relaxation of the masseter muscles directly after the massage. For this purpose, the visual analogue scale (VAS) was used. The VAS scale has been evaluated for orofacial pathologies [22]. In the present study, participants used the scale twice. First they rated their perceived pain, where 10 denoted the maximal pain and 0 denoted no pain, and next, they rated their perceived relaxation, where 10 denoted the maximal relaxation and 0 denoted no relaxation.

The data were statistically analysed and presented as means with standard deviations and medians with range. The Shapiro–Wilk test was used to test for normal distribution. Paired Student's t-test was used to compare the measurements before and after the massage. Unpaired Student's t-test was used to compare the measurements of the left and right masseters. Pearson's correlation coefficient was used to measure the strength of the association between elasticity values and the change in elasticity before and after the massage. Differences were considered statistically significant at *p* 0 < 0.05. Statistical analysis was carried out with the *R* Project for Statistical Computing v. 3.4.1.

## 3. Results

Overall, 21 subjects were enrolled in the study, but the data of 20 subjects (10 men and 10 women) were analysed. One participant withdrew written consent due to acute pain during the massage. The median age of the studied subjects was 34.5 years (range 18–60 years). The distributions of elasticity measurements were normal. The mean elasticity for both sides before the massage was 11.46 ± 1.55 kPa, and after the massage, it was 8.97 ± 0.96 kPa. The difference between the measurements was statistically significant (*p* < 0.0001). The mean difference before and after the massage was 2.49 ± 1.09 kPa. The drop in elasticity was observed in every patient. There was a strong positive correlation between the elasticity values before the massage and the difference between the two measurements (*r* = 0.79; *p* < 0.0001), which indicates that the drop in elasticity was greater in people with greater tonus. Such a correlation was not observed between after-massage measurements and the change in elasticity (*r* = 0.14; *p* = 0.3888). The detailed results are shown in Table 1. The intensity of pain reported by the studied subjects ranged between 6 and 9.5, with a median of 7.2 (men experienced a higher level of pain). All the subjects reported a sensation of relaxation directly after the massage, which was reflected on the VAS scale (median before massage 5.5 vs. median after massage 8.3). Also, elasticity values correlated significantly positively with the VAS score for relaxation after massage (*r* = 0.38; *p* = 0.0127).

**Figure 1:**
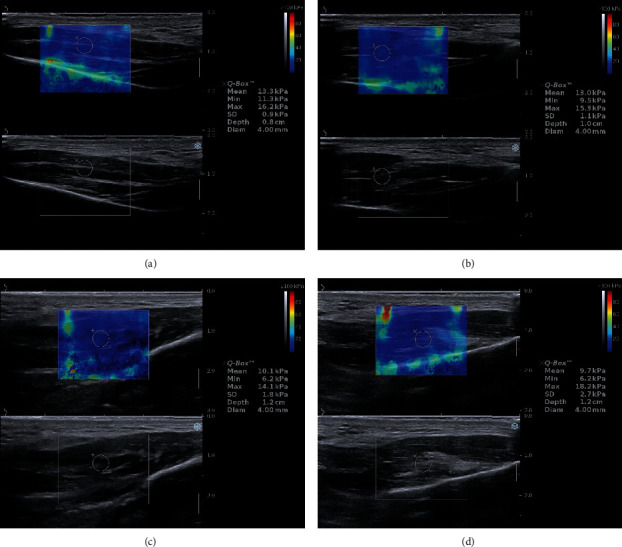
Measurements of elasticity in a 27-year-old female: (a) left masseter before massage; (b) right masseter before massage; (c) left masseter after massage; and (d) right masseter after massage.

Figure 1 shows the measurements performed on a 27-year-old female, a participant of the study group. The figure shows measurements of the elasticity of the left and right masseter muscle before and directly after the massage therapy. The images reveal a significant drop in stiffness values directly after the massage for all measurements. It corresponds to the sensation of relaxation of the muscle reported by the patient.

None of the subjects reported side effects or any unpleasant experience associated with the shear-wave elastography examination.

## 4. Discussion

Our study showed that shear-wave elastography reflects the changes in masseter muscle stiffness achieved by muscle massage. The elasticity of the masseter muscle dropped significantly from 11.46 ± 1.55 kPa before the massage to 8.97 ± 0.96 kPa after the massage. A decrease in elasticity was associated with an increase in the feeling of muscle relaxation reported by the subjects after the procedure. The shear-wave elastography examination was tolerated very well by all the participants of the study. To the best of our knowledge, this is the first study that investigates the response of the masseter muscle to massage that is measured with shear-wave elastography. The strength of this study lies in the fact that it proves that the subjective feeling of relaxation of the masticatory muscles after massage corresponds with the drop in stiffness observed using the objective shear-wave elastography method.

Massage applied to muscle tissue provides beneficial effects. It has been shown that massage reduces muscle stiffness, pain, and swelling; increases blood flow and the temperature of treated tissues; and exerts a general relaxation effect on the treated patient [23, 24]. However, the biomechanisms of the action of massage have not been fully elucidated. Despite these limitations, massage is a common form of physiotherapy used in a wide range of disorders [25]. Massage is also recommended for patients with TMD. In those patients, massage aims to revert the proper length flexibility of masticatory muscles. For this purpose, techniques such as effleurage, kneading, friction, and petrissage are widely used [17]. The present study evaluated 2 perceived feelings: (1) sensation of pain assessed using the VAS for pain during the massage and (2) sensation of relaxation after massage. The level of pain during the massage was high (median score of 7.2 out of 10), indicating that effective therapeutic massage requires application of pressure on trigger points. At the same time, this massage relieves tension. The subjects reported an increase in perceived relaxation of the masseter muscle (an increase from 5.5 to 8.3 scores).

Currently, no standards for the assessment of masseter muscle stiffness exist. Attempts to use shear-wave elastography to evaluate the effect of massage were reported in the literature. Eriksson Crommert et al. investigated the effect of a 7-minute massage of leg muscles in 18 healthy volunteers [26]. For the measurements, researchers used the Supersonic Aixplorer ultrasound scanner. A significant drop in elasticity was observed directly after massage in comparison to before massage, but the effect was no longer visible after 3 minutes, which suggested a short-term change. Moreover, there was no correlation between the rated pain level and a reduction in stiffness. Although elasticity values of leg muscles cannot be compared to those of the masseters, due to the fact that the values vary among different muscles [14], the study of Eriksson Crommert et al. brings valuable input into the assessment of the effect of massage on muscles using shear-wave elastography. Ariji et al. also carried out a series of studies on masseter muscle stiffness. In their study from 2009, different methods were used to those from our study [27]. First, the authors used strain elastography to evaluate stiffness, and therefore, the elasticity values cannot be compared due to the use of a different technique. Second, an oral rehabilitation robot was used for the massage. Finally, each person under study received five sessions of 1-minute robotic massage with various massage pressures. However, Ariji et al. came to an interesting conclusion that the masseter stiffness index correlated with the most comfortable massage pressure in healthy people. In another study by Ariji et al. from 2016, 37 patients with TMD suffering from myofascial pain were subjected to a 9.5-week massage therapy (five 16-minute sessions every 2 weeks) [11]. The researchers reported that only some of the patients responded to the treatment. This could be determined by the sonographic features of the masseter muscles. In massage participants, the median elasticity index ratios were decreasing gradually during the treatment, yet such a drop was not detected in those who did not participate.

The protocol of the shear-wave elastography of the masseter muscle still needs to be developed. In this study, we found that the most homogenous elasticity colour map was observed in the middle of the probe and cube box (colour-filled square, Figure 1). For this reason, we recommend taking measurements (round ROI) in the most homogenous field, which is in the middle of the square in almost all cases. We also recommend taking 3 separate measurements from 3 different images rather than, as was conducted in the present study, multiple measurements from 1 image. The method of measurement was modelled on previously published studies [16].

Despite the promising results, our study also has some limitations. We only evaluated the short-term response to massage. Long-term studies are needed, especially on patients with different types of masticatory muscle disorders, who could potentially benefit the most from the massage treatment. Furthermore, this study is a pre- and postmassage study showing the effectiveness of a single diagnostic technique, but in the future, comparative studies to evaluate shear-wave elastography in relation to other modalities are needed. In the present study, a 30-minute massage was conducted by a physiotherapist, while other researchers studied different forms of massage and much shorter session durations. The optimal massage protocol has not been established. Further research should focus on the selection of the most effective protocol. Shear-wave elastography, providing objective measurements, seems to be an important and valid tool for such an assessment. Moreover, we did not perform any reproducibility and interobserver variability, which requires further study. Finally, it may be valuable to extend the observation to other masticatory muscles (e.g., temporal) in order to investigate the impact of stiffness on patients suffering from TMD.

## 5. Conclusions

Our study showed that shear-wave elastography is a sensitive tool for monitoring changes in the stiffness of the masseter muscle after a single massage session. The objectivity and noninvasive character of this method and the provision of numerical values of stiffness seem to offer superiority with regards to previous methods and patient-reported effects. The potential of shear-wave elastography for use in clinical practice to monitor the condition of the masseter muscle should be further investigated in larger controlled studies.

## Figures and Tables

**Table 1:** Elasticity values before and after massage (data are shown in kPa).

	Before massage	After massage	*p*-value
Left masseter	11.55 ± 1.48	9.07 ± 1.00	<0.001
Right masseter	11.37 ± 1.66	8.87 ± 0.94	<0.001
*p*-value	0.7115	0.5291	

## Data Availability

The data used to support the findings of this study are included within the article.
